# Neutralizing antibody responses and cellular responses against SARS-CoV-2 Omicron subvariants after mRNA SARS-CoV-2 vaccination in kidney transplant recipients

**DOI:** 10.1038/s41598-024-63147-z

**Published:** 2024-05-28

**Authors:** Keita Kawashiro, Rigel Suzuki, Takuto Nogimori, Shuhei Tsujino, Naoya Iwahara, Takayuki Hirose, Kazufumi Okada, Takuya Yamamoto, Takasuke Fukuhara, Kiyohiko Hotta, Nobuo Shinohara

**Affiliations:** 1 Department of Urology, Hokkaido University Hospital, Sapporo, Japan; 2 Department of Microbiology and Immunology, Faculty of Medicine, Hokkaido University, Sapporo, Japan; 3 Institute for Vaccine Research and Development: HU-IVReD, Hokkaido University, Sapporo, Japan; 4 Laboratory of Precision Immunology, Center for Intractable Diseases and ImmunoGenomics, National Institutes of Biomedical Innovation, Health and Nutrition, Ibaraki, Japan; 5 Laboratory of Aging and Immune Regulation, Graduate School of Pharmaceutical Sciences, Osaka University, Suita, Japan; 6 Department of Virology and Immunology, Graduate School of Medicine, Osaka University, Suita, Japan; 7 Laboratory of Virus Control, Research Institute for Microbial Diseases, Osaka University, Suita, Japan; 8 Data Science Center, Promotion Unit, Institute of Health Science Innovation for Medical Care, Hokkaido University Hospital, Sapporo, Japan; 9 AMED-CREST, Japan Agency for Medical Research and Development (AMED), Tokyo, Japan

**Keywords:** Infection, Allotransplantation

## Abstract

Although the mRNA SARS-CoV-2 vaccine has improved the mortality rate in the general population, its efficacy against rapidly mutating virus strains, especially in kidney transplant recipients, remains unclear. We examined the anti-SARS-CoV-2 spike protein IgG antibody and neutralizing antibody titers and cellular immunity against B.1.1, BA.1, and BA.5 antigens in 73 uninfected kidney recipients and 16 uninfected healthy controls who received three doses of an mRNA SARS-CoV-2 vaccine. The IgG antibody titers were significantly lower in recipients than in healthy controls. Similarly, neutralizing antibody titers against three viral variants were significantly lower in recipients. When the virus was mutated, the neutralizing antibody titers decreased significantly in both groups. In cellular immunity analysis, the number of spike-specific CD8 + non-naïve T cells against three variants significantly decreased in recipients. Conversely, the frequency of spike-specific Th2 CD4 + T-cells in recipients was higher than that in healthy controls. Nineteen recipients and six healthy controls also received a bivalent omicron-containing booster vaccine, leading to increase IgG and neutralizing antibody titers in both groups. After that, eleven recipients and five healthy controls received XBB.1.5 monovalent vaccines, increasing the neutralizing antibody titers against not only XBB.1.5, but also EG.5.1 and BA.2.86 antigens in kidney recipients. Although kidney recipients did not gain sufficient immunity against Omicron BA.5 with the third dose of vaccine, humoral response against mutant SARS-CoV-2 lineages significantly increased after bivalent Omicron-containing booster vaccine and the XBB.1.5 monovalent vaccine. Therefore, it is important for kidney recipients to continue to administer updated vaccines.

## Introduction

Severe acute respiratory syndrome coronavirus 2 (SARS-CoV-2) infection was first identified in Wuhan, China, in 2019 and has since emerged as a pandemic. The mortality rate of SARS-CoV-2 infection before vaccination is higher in kidney transplant recipients (KTXRs) than in healthy controls (20–28% vs. 1–5%)^1^. Even after vaccination, KTXRs have a lower seroconversion rate, a higher infection rate, and severe disease^2,3^. The seroconversion rate in KTXRs after the third dose of the vaccine is 53–67%, and in healthy controls, it is almost 100%. KTXRs have more severe SARS-CoV-2 infection than healthy controls^3–7^. Furthermore, KTXRs have lower neutralizing antibody titers and cellular responses against B.1.1 (WT), B.1.617.2 (Delta), and B.1.1.529 (Omicron BA.1) after SARS-CoV-2 vaccination than healthy controls^8,9^. However, to our knowledge, few studies have evaluated neutralizing antibodies and cellular responses against Omicron BA.5 in KTXRs. Moreover, although the continuous evolution of SARS-CoV-2 and the rapid emergence of antigenically divergent variants has led to updates in the COVID-19 vaccine formulations, its efficacy against rapidly mutating virus strains, especially in KTXRs, remains unclear. In this study, we investigated the anti-SARS-CoV-2 spike protein IgG and neutralizing antibody titers and cellular responses against the Omicron subvariant BA.5 in KTXRs after the third dose of an mRNA vaccine. Furthermore, we evaluated the effects of a bivalent Omicron-containing booster and XBB.1.5 monovalent vaccination on KTXRs.

## Results

### Patient characteristics

The study included 73 KTXRs and 16 healthy controls who received up to three doses of the SARS-CoV-2 mRNA vaccine. Characteristics of the KTXRs and healthy controls are presented in Table 1. The median ages of KTXRs and healthy controls were 59 (IQR: 47–64) and 42 (IQR: 32–59) years, respectively (p = 0.0047). The median durations after the third vaccination in the KTXRs and healthy controls were 3.6 (IQR: 2.8–4.1) and 7.4 (IQR: 6.2–10.3) months, respectively (p < 0.0001).

**Table 1:** Baseline characteristics of kidney transplant recipients and healthy controls.

	KTXRs (n = 73)	Healthy (n = 16)	P value
Female, n (%)	40 (54.8)	5 (31.5)	0.0880
Age, years	59 (47–64)	42 (32–59)	0.0047
BMI, kg/m^2^	22.3 (20.7–25.4)	21.5 (19.1–23.3)	0.1253
Hypertension n (%)	48 (65.80)	1 (6.2)	< 0.0001
Diabetes mellitus n (%)	23 (31.5)	0 (0)	0.0091
Lymphocytes, 10^9^/L	1.2 (0.9–1.7)		
eGFR, mL/min/1.73m^2^	46.9 (38.2–54.1)		
Transplant characteristics			
First kidney transplant, n (%)	67 (91.8)		
Time after the last transplantation, years	4.1 (2.4–10.5)		
Living donor, n (%)	68 (93.2)		
Preemptive, n (%)	27 (37.0)		
ABO compatible, n (%)	54 (74.0)		
Number of immunosuppressive agents, n (%)			
2	6 (8.2)		
3	64 (87.7)		
4	3 (4.1)		
Immunosuppressive treatment, n (%)			
Steroids	68 (93.2)		
Mycophenolate mofetil	71 (97.3)		
Calcineurin inhibitor	71 (97.3)		
TAC/CYA	64 (87.7)/7 (9.6)		
mTOR inhibitor	5 (6.8)		
Time after the third SARS-CoV-2 vaccine, m	3.6 (2.8–4.1)	7.4 (6.2–10.3)	< 0.0001
Third vaccine makers, n (%)			
Pfizer	34 (46.6)	10 (62.5)	0.2486
Moderna	32 (43.8)	4 (25.0)	0.1644
Unknown	7 (9.6)	2 (12.5)	0.7540

Data are presented by frequency (%) or by median (IQR). P values are calculated using the nonparametric Mann–Whitney *U* test and chi-square test.

*KTXR* kidney transplant recipient, *BMI* body mass index, *eGFR* estimated glomerular filtration rate, *TAC* Tacrolimus, *CYA* Cyclosporine A.

### Anti-SARS-CoV-2 spike protein IgG antibody production by SARS-CoV-2 vaccination in KTXRs

**Figure 1:**
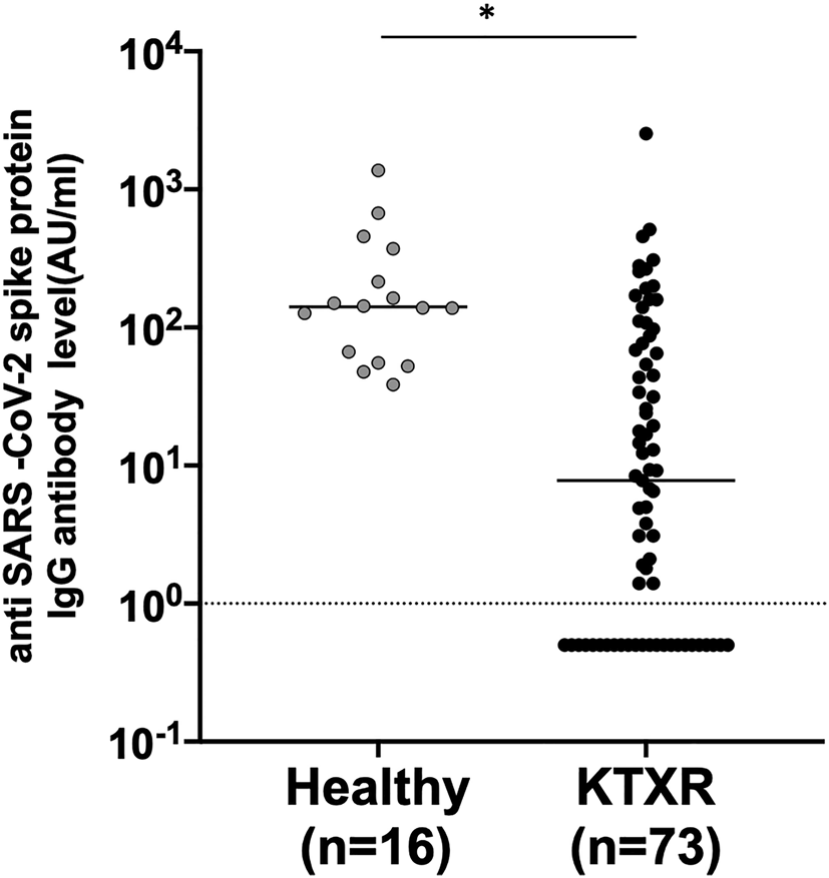
Anti-SARS-CoV-2 spike protein IgG antibody titers in healthy controls and KTXRs. Anti-SARS-CoV-2 spike protein IgG antibody titers in 16 healthy controls and 73 KTXRs. The cut-off value was defined as 1.0 AU/mL. *P < 0.0001, *P*-values were calculated using the nonparametric Mann–Whitney *U* test. KTXR, kidney transplant recipient.

Anti-SARS-CoV-2 spike protein IgG antibody titers in both groups are shown in Fig. 1. The median antibody titers in the KTXRs and healthy controls after the third dose of vaccine were 7.8 (IQR: < 1–72.7) and 141.0 (IQR: 58.3–332.5) AU/mL respectively, and the antibody titers in the KTXRs were significantly lower than those in healthy controls (p < 0.0001). The antibody positivity rates were 67.1% and 100%. The background factors for the responders and non-responders among KTXRs are summarized in Table 2. The time after kidney transplantation was significantly longer in the responder group than in the non-responder group (p = 0.0012). Furthermore, the mycophenolic acid area under the plasm concentration–time curve was significantly lower in responders than in non-responders (p = 0.0041). No significant differences were observed in the other background factors.

**Table 2:** Differences in subject characteristics between responders versus non-responders among kidney transplant recipients.

	Responder (n = 49)	Non-responder (n = 24)	P value
Female, n (%)	26 (53.1)	14 (58.3)	0.6707
Age, years	58 (47–64)	59 (48–67)	0.6850
BMI, kg/m^2^	22.7 (20.1–25.9)	22.2 (20.8–23.8)	0.5935
Lymphocytes, 10^9^/L	1.2 (1.0–1.7)	1.0 (0.8–1.4)	0.2120
eGFR, mL/min/1.73 m^2^	48.7 (40.0–54.6)	41.9 (31.2–52.7)	0.1195
Transplant characteristics			
First kidney transplant, n (%)	46 (93.9)	21 (87.5)	0.3513
Time after the last transplantation, years	6.4 (2.9–11.7)	3.0 (1.5–6.1)	0.0012
Last transplant			
Living, n (%)	45 (91.8)	23 (95.8)	0.5254
Preemptive, n (%)	20 (40.8)	7 (29.2)	0.3328
ABO compatible, n (%)	36 (73.5)	18 (75.0)	0.8887
Number of immunosuppressive agents	3 (3–3)	3 (3–3)	0.0593
Immunosuppressive treatment, n (%)			
Steroids	44 (89.8)	24 (100.0)	0.1049
Mycophenolate mofetil	47 (95.9)	24 (100.0)	0.3156
Calcineurin inhibitor	47 (95.9)	24 (100.0)	0.3156
mTOR inhibitor	3 (6.1)	2 (8.3)	0.7254
TAC Trough, ng/mL^a^	3.0 (2.5–3.8)	3.2 (2.1–3.6)	0.7943
MPA AUC, mg⋅h/L^b^	45.0 (35.0–56.7)	57.7 (47.6–71.4)	0.0027
Time after the third SARS-CoV-2 vaccine, m	3.7 (2.9–4.1)	3.4 (1.2–3.7)	0.3613
Third vaccine makers, n (%)			
Pfizer	20 (40.8)	14 (58.3)	0.1587
Moderna	23 (47.0)	9 (37.5)	0.4425
Unknown	6 (12.2)	1 (4.2)	0.2308

Data are presented as frequency (%) or median (IQR). P values are calculated using the nonparametric Mann–Whitney *U* test and chi-square test.

*TAC* tacrolimus, *MPA AUC* mycophenolic acid area under the curve.

^a^TAC Trough levels are available in 37 responders and 22 non-responders.

^b^MPA-AUC levels are available in 43 responders and 24 non-responders.

### Neutralizing activity of sera against SARS-CoV-2 variants after the third vaccine dose

**Figure 2:**
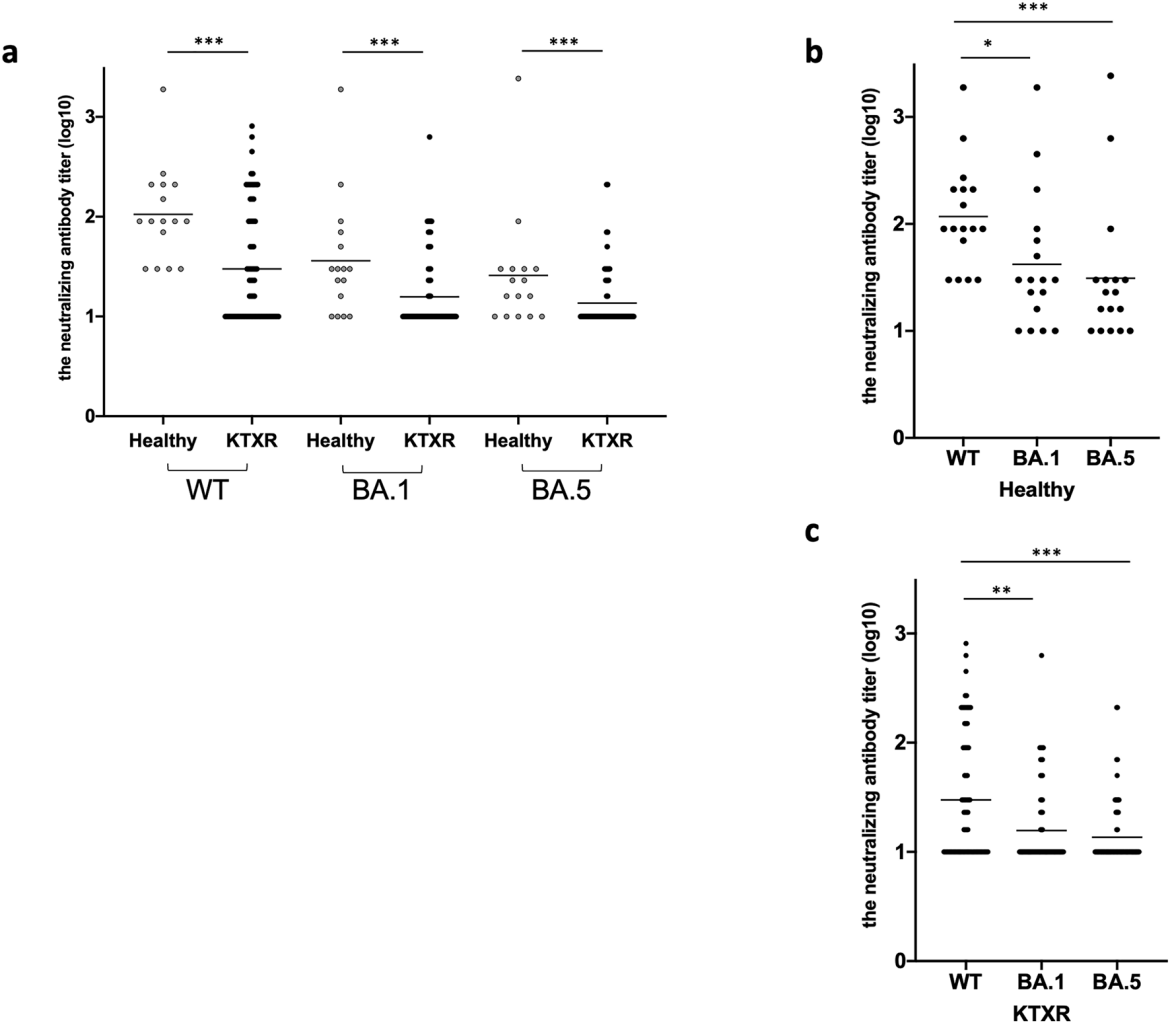
Neutralizing activity against SARS-CoV-2 variants in healthy controls and KTXRs after the third vaccine dose. (**a**) The neutralizing antibody titer (log10) against rWT S-GFP, rBA.1 S-GFP, and rBA.5 S-GFP in healthy controls and KTX recipients after the third dose of mRNA vaccine. The chimeric recombinant SARS-CoV-2 and diluted serum mixture was inoculated into VeroE6/TMPRSS2 cells. After post-infection, the expression of GFP in VeroE6/TMPRSS2 cells was observed by fluorescent microscopy and the titer of the neutralizing antibody was calculated. ***P < 0.0001, *P*-values were calculated using the nonparametric Mann–Whitney *U* test. (**b**) The neutralizing antibody titer (log10) against rWT S-GFP, rBA.1 S-GFP, and rBA.5 S-GFP in healthy controls. This data was extracted from (**a**). *P < 0.05, Dunn’s multiple-comparison method was used to test for significant differences among the three groups. (**c**) The neutralizing antibody titer (log10) against rWT S-GFP, rBA.1 S-GFP, and rBA.5 S-GFP in KTXRs. This data was extracted from (**a**). **P < 0.01, ***P < 0.0001, Dunn’s multiple-comparison method was used to test for significant differences among the three groups. KTXR, kidney transplant recipient.

To measure the neutralizing activity of sera from KTXRs against B.1.1 (WT), BA.1, and BA.5, we generated green fluorescent protein (GFP)-carrying recombinant SARS-CoV-2 with spike protein of WT, BA.1, or BA.5 by reverse genetics (rWT S-GFP, rBA.1 S-GFP, and rBA.5 S-GFP, respectively). Using these chimeric recombinant viruses, we performed a high-throughput neutralization assay evaluated by visual examination of the GFP signal. We previously compared the 50% neutralizing titer (NT_50_) calculated by reverse transcription-quantitative polymerase chain reaction (RT-qPCR) and neutralizing activity titer calculated by visual measurement of GFP expression using a fluorescent microscope. The NT_50_ calculated from viral RNA levels in the supernatants of infected cells was well correlated with the titer of neutralizing antibodies calculated by GFP fluorescence intensity^10,11^. The neutralizing antibody titers against rWT S-GFP, rBA.1 S-GFP, and rBA.5 S-GFP in KTXRs and healthy controls after the third dose of the mRNA vaccine were shown in Fig. 2a. The neutralizing antibody titers against all types of viruses in KTXRs were significantly lower than those in healthy controls (p < 0.001). Interestingly, when the virus was mutated, the neutralizing antibody titers in both groups were significantly decreased (Fig. 2b and c). These results indicate that rBA.1 and rBA.5 S-GFP evade neutralizing antibodies in healthy controls and KTXRs.

### Induction of spike-specific T-cell responses in healthy donors and KTXRs

**Figure 3:**
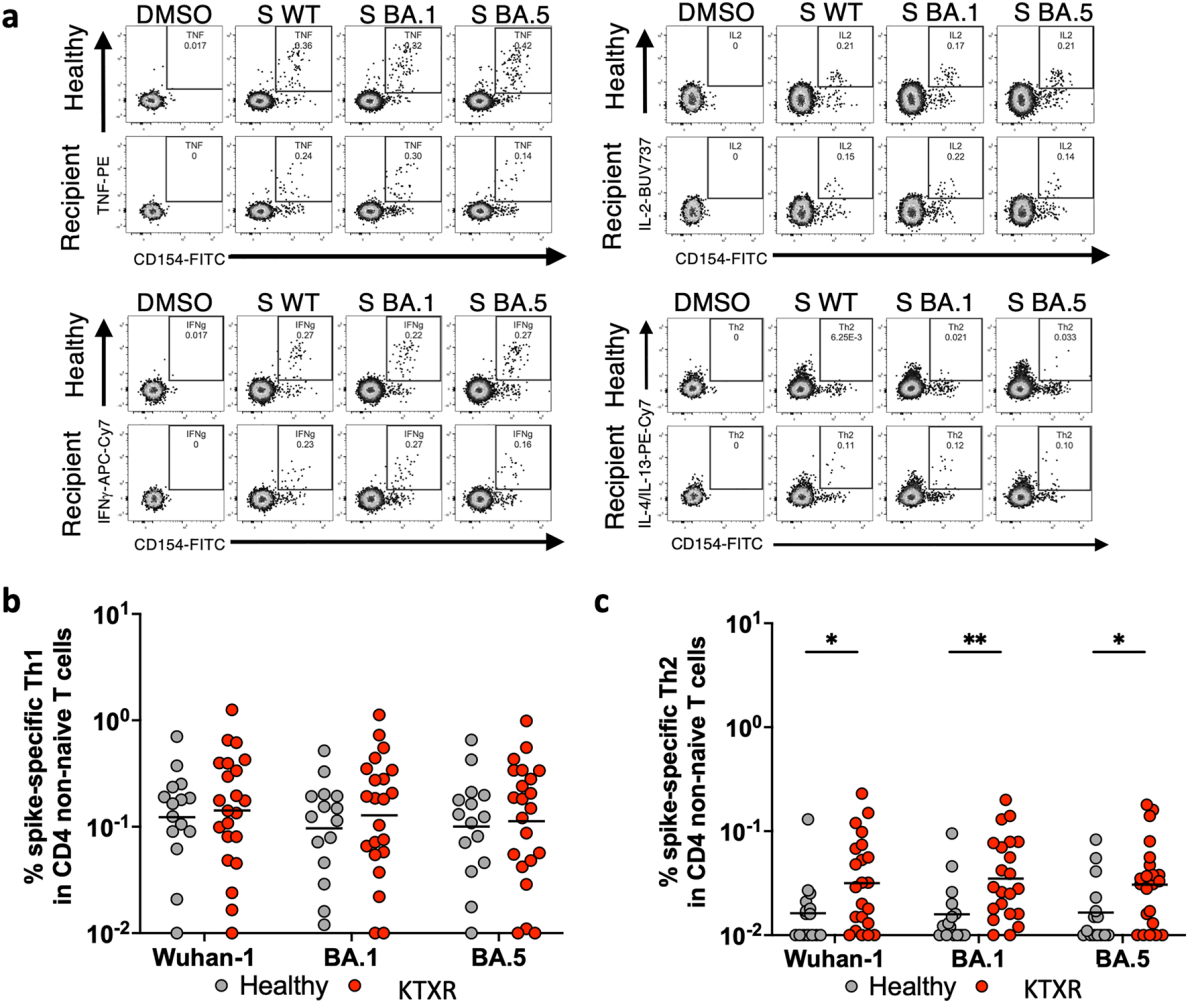
Spike-specific CD4 + T-cell responses in healthy controls and KTXRs. (**a**) After gating live single T cells, based on forward scatter area and height (FSC-A and -H), side scatter area (SSC-A), live/dead cell exclusion, and CD3 staining, we separated the PBMCs into CD4 + . Subsequently, CD4 + T cells were further divided into non-naïve phenotypes based on the expression of CD27 and CD45RO. We defined CD154 + CD4 T cells expressing IFN-γ (upper left panels), TNF (upper right panels), or IL-2 (bottom left panels) as Th1 cells and expressing IL4 or IL-13 (bottom right panels) as Th2 cells. (**b**) The frequency of spike-specific Th1 CD4 T cells against Wuhan-1 (Healthy controls; 0.01–0.704%, geometric mean = 0.123%, KTXRs; 0.01–1.254%, geometric mean = 0.142%), BA.1 (Healthy controls; 0.012–0.517%, geometric mean = 0.097%, KTXRs; 0.01–1.123%, geometric mean = 0.128%), or BA.5 (Healthy controls; 0.01–0.654%, geometric mean = 0.1%, KTXRs; 0.01–0.986%, geometric mean = 0.113%) in CD4 non-naïve T cells from vaccinated healthy controls and KTXRs. *P*-values were calculated using the nonparametric Mann–Whitney *U* test. (**c**) The frequency of spike-specific Th2 CD4 T cells against Wuhan-1 (Healthy controls; 0.01–0.13%, geometric mean = 0.016%, KTXRs; 0.01–0.23%, geometric mean = 0.032%), BA.1 (Healthy controls; 0.01%-0.095%, geometric mean = 0.016%, KTXRs; 0.01–0.2%, geometric mean = 0.035%), or BA.5 (Healthy controls; 0.01%-0.083%, geometric mean = 0.016%, KTXRs; 0.01–0.18%, geometric mean = 0.031%) in CD4 non-naïve T cells from vaccinated healthy controls and KTXRs. *P < 0.05, **P < 0.01, and *P*-values were calculated using the nonparametric Mann–Whitney *U* test.

Immunosuppressive drugs, including calcineurin inhibitors, generally suppress T cell activity. We performed flow cytometry analysis to investigate whether spike-specific CD4 + T cell responses in KTXRs were induced by mRNA vaccines. Peripheral blood mononuclear cells (PBMCs) obtained from 22 KTXRs and 15 healthy controls were stimulated with overlapping peptides corresponding to the SARS-CoV-2 spike protein. We defined CD154 + CD4 + T cells expressing IFN-g, TNF, or IL-2 as Th1 cells and those expressing IL4 or IL-13 as Th2 cells (Fig. 3a)^12–14^. Spike-specific Th1-cell and Th2-cell frequencies were determined in the stimulated samples by subtracting the background signal observed in the unstimulated samples. The frequency of spike-specific Th1 cells against WT spikes in KTXRs was comparable to that in healthy controls (Fig. 3b, p = 0.7314). Furthermore, mRNA vaccine-induced spike-specific Th1 CD4 T-cell responses against BA.1 and BA.5 were at comparable levels to those of Th1 CD4 T-cell responses against WT in both healthy controls and KTXRs. In contrast, the frequency of spike-specific Th2 cells against WT, BA.1, and BA.5 spikes in KTXRs was higher than that in healthy controls (Fig. 3c, WT: p = 0.0312, BA.1: p = 0.0037, BA.5: p = 0.0474). These results indicate that mRNA vaccines can induce spike-specific CD4 + T- cell responses to omicron sublineages in healthy controls and in KTXRs. Besides, KTXRs are more susceptible to the induction of Th2-biased CD4 + T cell responses.

**Figure 4:**
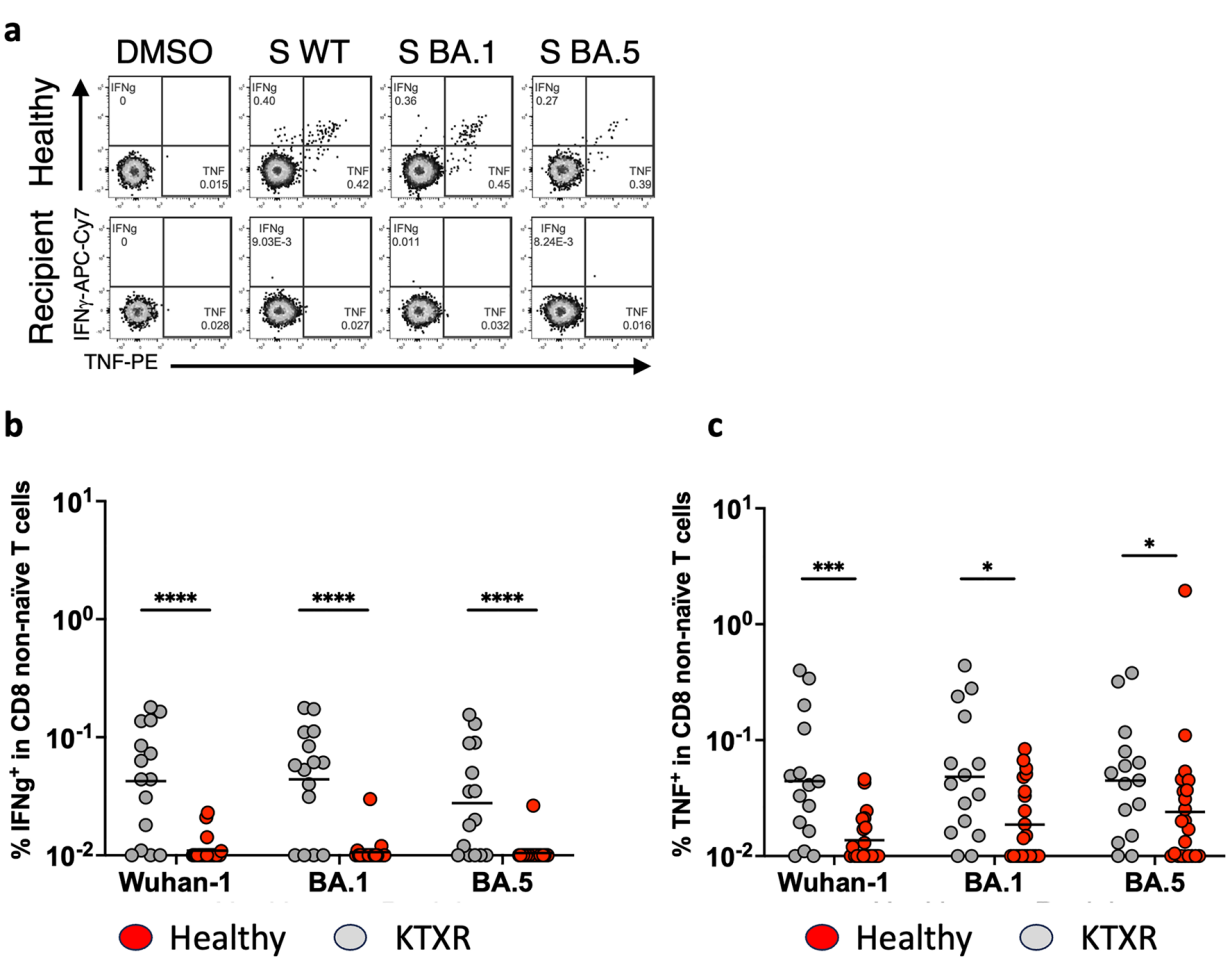
Spike-specific CD8 + T-cell responses in healthy controls and KTXRs. (**a**) After gating live single T cells, based on forward scatter area and height (FSC-A and -H), side scatter area (SSC-A), live/dead cell exclusion, and CD3 staining, we separated the PBMCs into CD8 + T cells. Subsequently, CD8 + T cells were further divided into non-naïve phenotypes based on the expression of CD27 and CD45RO. For spike-specific CD8 T cells, non-naïve cells were gated based on the expression of IFN-γ or TNF. (**b**) The frequency of spike-specific CD8 T cells producing IFN-γ against Wuhan-1 (Healthy controls; 0.01–0.18%, geometric mean = 0.042%, KTXRs; 0.01–0.023%, geometric mean = 0.011%), BA.1 (Healthy controls; 0.01–0.177%, geometric mean = 0.044%, KTXRs; 0.01–0.03%, geometric mean = 0.011%), or BA.5 (Healthy controls; 0.01–0.155%, geometric mean = 0.028%, KTXRs; 0.01–0.026%, geometric mean = 0.01%) in CD8 t non-naïve T cells from vaccinated healthy controls and KTXRs. ****P < 0.0001, *P*-values were calculated using the nonparametric Mann–Whitney *U* test. (**c**) The frequency of spike-specific CD8 T cells producing TNF against Wuhan-1 (Healthy controls; 0.01–0.4%, geometric mean = 0.044%, KTXRs; 0.01–0.046%, geometric mean = 0.014%), BA.1 (Healthy controls; 0.01–0.44%, geometric mean = 0.048%, KTXRs; 0.01–0.084%, geometric mean = 0.019%), or BA.5 (Healthy controls; 0.01–0.38%, geometric mean = 0.045%, KTXRs; 0.01–1.95%, geometric mean = 0.024%) in CD8 non-naïve T cells from vaccinated healthy controls and KTXRs. *P < 0.05, ***P < 0.001, and *P*-values were calculated using the nonparametric Mann–Whitney *U* test. *KTXR* kidney transplant recipient.

In addition to antibodies and CD4 + T-cell responses, spike-specific CD8 + T-cell responses contribute to defense against SARS-CoV-2 infection^15–18^. Subsequently, we examined whether CD8 + T cell responses were induced by mRNA vaccines in KTXRs using flow cytometry. Spike-specific CD8 + T cells were gated based on the expression of IFN-g or TNF in total non-naïve CD8 + T cells (Fig. 4a). The frequencies of spike-specific CD8 + T cells producing IFN-g and TNF against WT, BA.1, and BA.5 spikes in KTXRs were lower than those in healthy controls (Fig. 4b, WT: p < 0.0001, BA.1: p < 0.0001, BA.5: p < 0.0001 and Fig. 4c, WT: p = 0.0005, BA.1: p = 0.011, BA.5: p = 0.0405). These results indicate that immunosuppressive drugs potentially affect spike-specific CD8 + T-cell responses in KTXRs.

### Neutralizing activity of sera against rBA.5 S-GFP virus after administering the bivalent Omicron-containing booster vaccine

**Figure 5:**
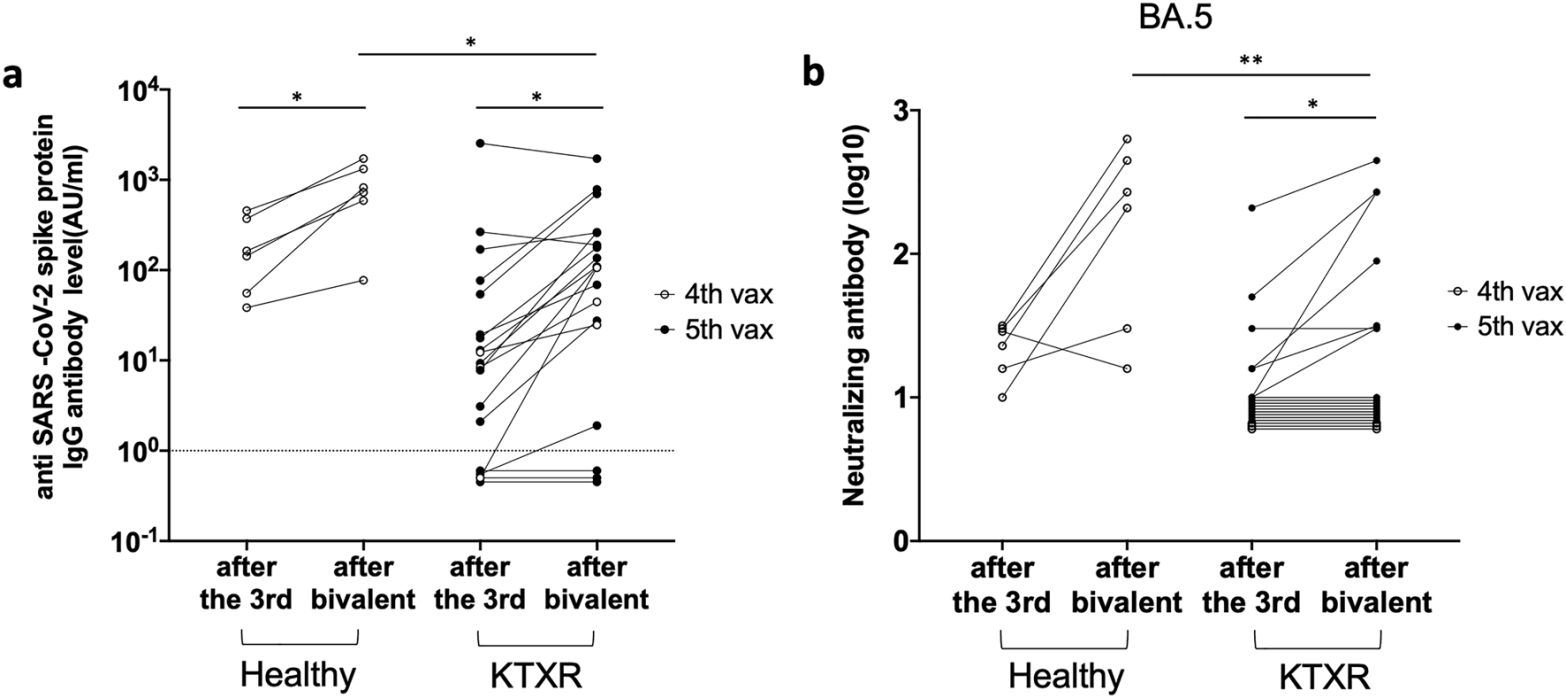
Anti-SARS-CoV-2 spike protein IgG antibody titers and the neutralizing activity titers against rBA.5 S-GFP virus in healthy controls and KTXRs after a third vaccine dose and bivalent omicron-containing booster vaccine. (**a**) Anti-SARS-CoV-2 spike protein IgG antibody titers in healthy controls and KTXRs after a third vaccine dose and bivalent omicron-containing booster vaccine. *P < 0.05, *P*-values were calculated using the Wilcoxon signed-rank test. (**b**) The neutralizing antibody titer (log10) against rBA.5 S-GFP virus in healthy controls and KTXRs after a third vaccine dose and bivalent omicron-containing booster vaccine. The mixture of rBA.5 S-GFP virus and diluted serum of healthy controls or KTXRs was inoculated into VeroE6/TMPRSS2 cells. After post-infection, the expression of GFP in VeroE6/TMPRSS2 cells was observed by fluorescent microscopy, and the titer of the neutralizing antibody was calculated. *P < 0.05, **P < 0.01, and *P*-values were calculated using the Wilcoxon signed-rank test. KTXR, kidney transplant recipient.

Characteristics of the KTXRs and healthy controls are presented in Table S1. Among the 19 KTXRs who received the bivalent omicron-containing booster vaccine, three received it after the third dose and 16 after the fourth dose. All six healthy controls received the bivalent omicron-containing booster vaccine after the third dose. We measured the anti-SARS-CoV-2 spike protein IgG and anti-N IgG antibody titers. Furthermore, we performed a neutralizing antibody assay using the rBA.5 S-GFP virus. No KTXRs or healthy controls developed anti-N IgG antibodies after the third and bivalent omicron-containing booster vaccination. This result suggests that the specimens used in this study have never been infected with SARS-CoV-2. The anti-SARS-CoV-2 spike protein IgG antibody titers and neutralizing activity of both groups are shown in Fig. 5. Anti-SARS-CoV-2 spike protein IgG antibody titers were significantly elevated in both groups (Fig. 5a, recipients: p = 0.0155, healthy controls: p = 0.0312). However, antibody titers after the bivalent vaccine in healthy controls were significantly higher than those in KTXRs (Fig. 5a, P = 0.0177). Although the neutralizing activity was significantly elevated in KTXRs (Fig. 5b, p = 0.0312), the neutralizing activity was higher in healthy controls than in KTXRs after the bivalent vaccine (Fig. 5b, P = 0.0094).

### Neutralizing activity of sera against XBB.1.5, EG.5.1, and BA2.86 after XBB.1.5 monovalent vaccination

**Figure 6:**
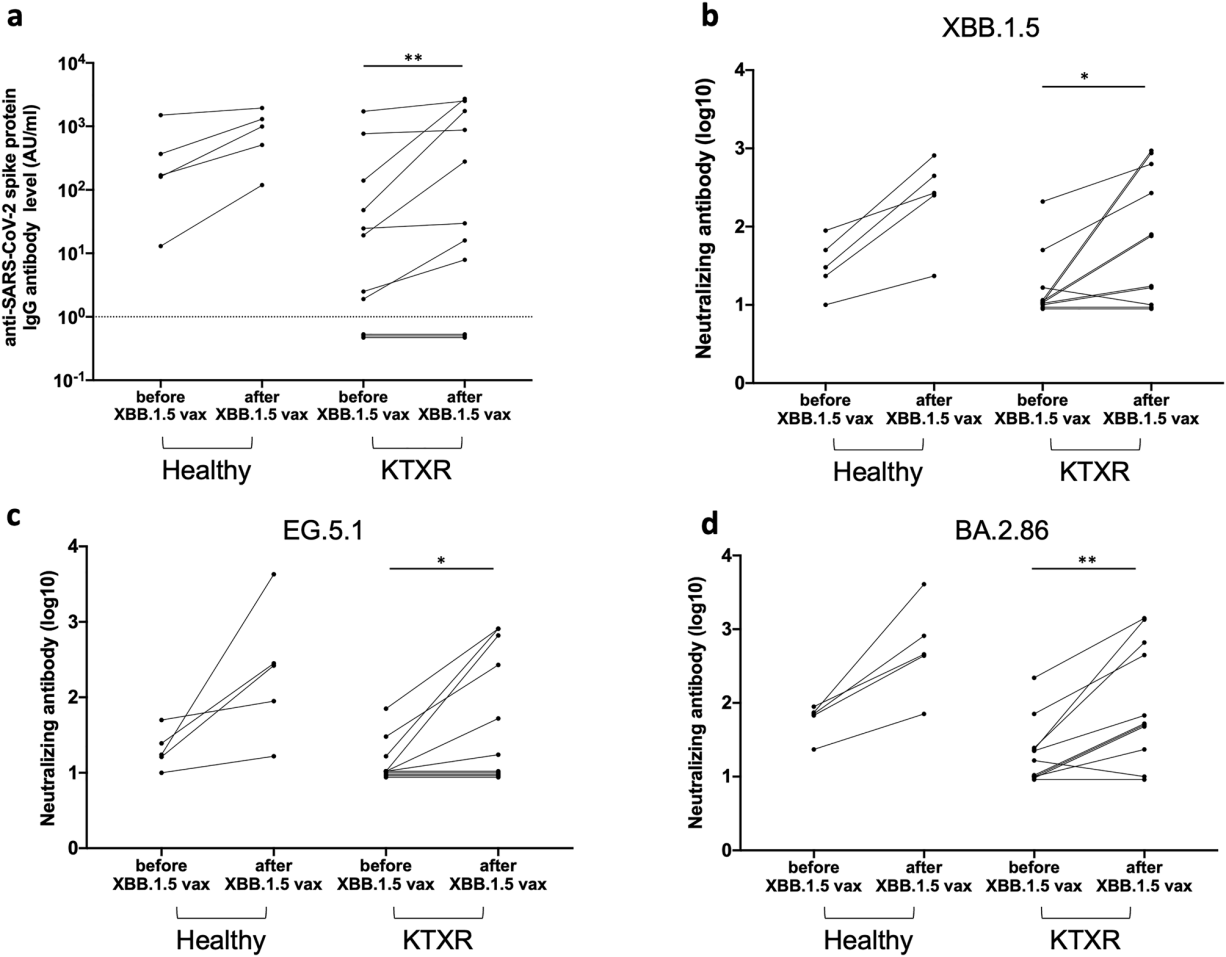
Anti-SARS-CoV-2 spike protein IgG antibody titers and the neutralizing antibody titers against rXBB.1.5, rEG.5.1 and rBA.2.86 in healthy controls and KTXRs before and after XBB.1.5 monovalent vaccination. (**a**) Anti-SARS-CoV-2 spike protein IgG antibody titers in healthy controls and KTXRs before and after XBB.1.5 monovalent vaccination. **P < 0.01, *P*-values were calculated using the Wilcoxon signed-rank test. (**b**–**d**) The neutralizing antibody titer (log10) against (**b**) rXBB.1.5, (**c**) rEG.5.1, (**d**) rBA.2.86 in healthy controls and KTXRs before and after XBB.1.5 monovalent vaccination. The chimeric recombinant SARS-CoV-2 and diluted serum mixture was inoculated into VeroE6/TMPRSS2 cells. Post-infection, the expression of GFP in VeroE6/TMPRSS2 cells was observed by fluorescent microscopy and the titer of the neutralizing antibody was calculated. *P < 0.05, *P*-values were calculated using the Wilcoxon signed-rank test.

Characteristics of the KTXRs and healthy controls are presented in Table S2. No KTXRs or healthy controls developed anti-N IgG antibodies before and after XBB.1.5 monovalent vaccination. Anti-SARS-CoV-2 spike protein IgG antibody titers increased significantly after XBB.1.5 monovalent vaccination in KTXRs (p = 0.0078). The antibody titers increased in all healthy controls after XBB.1.5 monovalent vaccination (Fig. 6a). The neutralizing antibody titers against rXBB.1.5-GFP, rEG.5.1-GFP, and rBA.2.86-GFP in KTXRs and healthy controls before and after XBB.1.5 monovalent vaccination are shown in Fig. 6b–d. The neutralizing activity against each variant in KTXRs was significantly elevated (rXBB.1.5-GFP: p = 0.0156, rEG.5.1-GFP: p = 0.0312, rBA.2.86-GFP: p = 0.0039). All healthy controls showed an increase of the neutralizing activity against each variant.

## Discussion

To our knowledge, this is the first study to focus on a single cohort of KTXRs and to investigate the neutralizing antibody titers and T-cell responses against SARS-CoV-2 Omicron BA.5 after a third dose of an mRNA SARS-CoV-2 vaccine, a bivalent omicron-containing booster vaccine, and an XBB.1.5 monovalent booster vaccine. Although KTXRs did not gain sufficient immunity against Omicron BA.5 with the third dose of the vaccine, humoral response against mutant SARS-CoV-2 lineages was significantly increased by the bivalent Omicron-containing booster vaccine and XBB.1.5 monovalent vaccine. Although similar studies have reported comprehensive analysis in solid organ transplant recipients (kidney, liver, heart, lung, and multiple organs), there are few studies in a single cohort of KTXRs^19–23^. Since the immunosuppression status in solid organ recipients differs depending on their type of graft organ, analysis of immune responses only in KTXRs is useful for future COVID-19 infection control in KTXRs.

Recent studies have reported that the COVID-19 severity of the Omicron variants is less than that of the previous lineages in both the general population and KTXRs^24–28^. However, Solera et al. reported that the rate of hospitalization for COVID-19 in solid organ transplant recipients (SORTs) was 9.8% during the XBB.1.5 phase, which was higher than that in general population, suggesting that KTXRs might remain susceptible to current Omicron lineages and develop more severe disease^29,30^. Therefore, the development of an effective prophylaxis strategy for COVID-19 infection is essential in KTXRs.

First, we evaluated the anti-SARS-CoV-2 spike protein IgG antibody levels and detected seropositivity in 67.1% of the KTXRs after the third vaccination. Similarly, Benning et al. reported an antibody-positive rate of 71% in KTXRs 3 weeks after the third vaccination^8^. Al Jurdi et al. reported that the percentage of KTXRs with anti-SARS-CoV-2 spike protein IgG antibodies increased from 29 to 67% after a third vaccination^6^. Therefore, antibody titers were increased after the third vaccination but were considerably lower than those in healthy controls. We demonstrated that the time after kidney transplantation was significantly shorter in the non-responder group than in the responder group. Higher risk may be primarily attributed to higher levels of immunosuppression early on, shortly after kidney transplantation. A high mycophenolate mofetil (MMF) blood concentration was significantly associated with the risk of being a non-responder. In contrast, the tacrolimus trough concentration was similar between the two groups. A large cohort study (N = 1804) also reported similar findings^31^. Several studies demonstrated that IgG positivity is significantly associated with MMF cessation or dose reduction^32–34^. Tomiyama et al. reported that the seropositivity rate was 95% after the third dose of the vaccine in liver transplant recipients, and only 48% among them were taking MMF^10^. Netti et al. reported that mTOR inhibitors were beneficial in mRNA vaccine-induced immunogenicity^35^. However, Bae et al. concluded that MMF avoidance, rather than mTOR inhibitors use, is associated with improved SARS-CoV-2 mRNA vaccine responses in solid organ transplant recipients^36^. These studies indicated that MMF strongly contributes to the suppression of antibody production in organ transplant recipients. In contrast, there are several papers that critically evaluate the effects of MMF withdrawal on improving vaccine responsiveness. Regele et al. reported that MMF cessation in KTXRs did not increase the response to SARS-CoV-2 vaccination^37^. Kühn et al. reported that the initial effect of enhanced seroconversion rates in KTXRs with MMF withdrawal was limited due to the fact that the effect disappearing 3 months after vaccination^38^. Furthermore, Benning et al. reported that although no rejection occurred during MMF withdrawal, a resurgence of prior anti-HLA donor-specific antibodies was detected^39^. Therefore, MMF withdrawal to improve vaccine responsiveness should be carefully considered.

We showed that the neutralizing titers against Omicron BA.5 in KTXRs were significantly lower than those in healthy controls using the live SARS-CoV-2 virus neutralization assay, indicating that recipients are at a higher risk of infection and that the vaccine is less effective in KTXRs. Werbel et al. reported similar results in a cohort of KTXRs (N = 81)^40^. Furthermore, we showed that neutralizing titers also decreased in KTXRs as the virus showed mutations from WT to BA.1 to BA.5. Tuekprakhon et al. reported that as the mutation evolved, neutralizing titers decreased in the general population after three vaccine doses^41^. Pedersen et al. reported the neutralizing titers after a third dose of vaccination in KTXRs decreased as the virus mutated from B.1.617.2 (delta) to BA.1 to BA.5^42^. These results suggests that KTXRs are at a higher risk of infection as the virus evolves.

T-cell responses have been shown to reduce severe illnesses caused by SARS-CoV-2. KTXRs are at a risk of severe SARS-CoV-2 infection because of their reduced T-cell immunity^43^. A previous study indicated that the third mRNA dose induced a T cell response against WT spike peptides in KTXRs, while cellular immunity toward Omicron BA.1 variant immunogenic peptides was low in both KTXRs and healthy controls^9^. However, no studies have evaluated the T-cell responses to Omicron BA.5 spike peptides. The frequency of spike-specific Th1 cells against BA.5 spike in KTXRs was comparable to that in healthy controls; however, Th2 CD4 + T cell responses were significantly higher in KTXRs. This result is unexpectedly interesting, and several studies describe similar phenomenon. Miyaura et al. reported that steroids inhibit the differentiation of CD4 T cells into Th1 while promoting differentiation into Th2 in vitro^44^. Another study examined the effects of MMF on Th1/Th2 differentiation in a Crohn’s disease mouse model^45^. The results indicated that MMF significantly suppressed the mRNA expression of the inflammatory cytokines IFN-g and TNF-a, typically associated with Th1 cells, in this model. However, MMF did not inhibit the expression of IL-10 mRNA, a cytokine associated with Th2 responses. Based on these reports, our findings indicate that immunosuppressants affect the differentiation of CD4 T cells in vitro, however, verification in humans in vivo/ex vivo is lacking. The Th1/Th2 balance of CD4 + T cells is crucial in cellular responses, and skewed distribution towards Th2 cells increases the risk of severe infection and vaccine-associated enhanced respiratory disease^46,47^. Further investigation is needed to evaluate the potential risks of increasing Th2 CD4 + T-cell responses in KTXRs.

The number of spike-specific CD8 + T cells producing IFN-g and TNF was significantly lower in KTXRs than in healthy controls. These results suggest that KTXRs did not acquire sufficient immunity against Omicron BA.5 (neither humoral nor in cellular immunity), even after receiving three vaccine doses^17,46,48^. Furthermore, we evaluated the effects of a bivalent Omicron-containing booster vaccine in KTXRs. Although the current bivalent Omicron-containing booster vaccine produced increased anti-SARS-CoV-2 spike protein IgG and neutralizing antibody titers, these effects were lower in KTXRs than in healthy controls. Fernández-Ruiz et al. reported that the neutralizing antibody responses against the BA.4/5 in SORTs increased after a bivalent Omicron-containing booster vaccine. However, booster-induced cell-mediated immunity remained lower when compared with healthy controls^23^. Yau et al. reported that the neutralizing antibody responses in dialysis patients and KTXRs increased significantly against not only BA.5 but also XBB.1.5 after a bivalent Omicron-containing booster vaccine; however, the neutralizing antibodies against XBB.1.5 was 5.8-fold lower than against BA.5 due to high immune evasion^49^. Recent studies have reported the effect of XBB.1.5 monovalent vaccines in the general population. The XBB.1.5 vaccine strongly increased the anti-SARS-CoV-2 spike protein IgG and elicited potent neutralizing responses against previous and contemporary SARS-CoV-2 lineages, including XBB.1.5, EG.5.1, and BA.2.86^50,51^. We investigated a similar effect of XBB.1.5 monovalent vaccines among KTXRs. This is the first study to analyze the anti-SARS-CoV-2 spike protein IgG and neutralizing antibody titers against XBB.1.5, EG.5.1, and BA.2.86 in KTXRs after XBB.1.5 monovalent vaccination. These data suggested the best prophylaxis strategy against COVID-19 at this time is to continue to administer updated vaccines in KTXRs.

As an alternative prophylaxis strategy, French researchers have reported the efficacy of tixagevimab/cilgavimab in reducing the morbidity and severity of SARS-CoV-2 in KTXRs who failed to develop a protective humoral response after at least three doses of an mRNA vaccine administration during the Omicron BA.1-BA.2 epidemic^52,53^. Recent clinical studies suggested that tixagevimab/cilgavimab improved the neutralization of BA.4/5, but this effect was not observed against BQ.1.1 and XBB.1.5^54,55^. Therefore, the effect of monoclonal antibody as prophylaxis for COVID-19 infection may be limited.

The strength of our study is the analysis of neutralizing activity using the live SARS-CoV-2 virus neutralization assay. Furthermore, this is the first study investigating T cell responses against SARS-CoV-2 Omicron BA.5 and the effect of bivalent Omicron-containing booster vaccines and XBB.1.5 monovalent booster vaccines exclusively in a kidney recipient cohort. Nonetheless, there are some limitations of our study. First, the time between vaccination and blood collection varied because the blood samples were collected during outpatient visits. Although KTXRs had a shorter term than controls, KTXRs had lower neutralizing activity. Therefore, the timing of sample collection did not significantly affect the results. Second, healthy controls are significantly younger than KTXRs. Several studies demonstrated that the antibody responses elicited by the mRNA SARS-CoV-2 vaccine declined with increasing vaccine age^56,57^. However, no statistical correlation between age and antibody titer or neutralizing antibody titers was observed in this cohort (Fig. S1). Therefore, the current study provided rational results, though comparing groups by propensity score matching would be ideal.

## Conclusion

The adaptive immunity of KTXRs was not significantly developed after a third dose of an mRNA vaccine, suggesting that recipients are at a higher risk for infection and severe disease than healthy controls. However, humoral response against mutant SARS-CoV-2 lineages significantly increased after the bivalent Omicron-containing booster and XBB.1.5 monovalent vaccines. Therefore, it is important for kidney recipients to continue to receive updated vaccines.

## Methods

### Study design and patients

We retrospectively reviewed the data of 403 recipients who underwent kidney transplantation at Hokkaido University between 1996 and 2021. Our inclusion criteria comprised patients who were administered up to three mRNA vaccines after kidney transplantation. Conversely, the following criteria were used to exclude patients from the study: (1) loss of graft function, (2) SARS-CoV-2 infection before initiation of the study and (3) development of anti-N IgG antibody before initiation of the study. Therefore, 73 patients were included in the final analysis. As healthy controls, we reviewed the data from seven kidney donors who underwent nephrectomy at Hokkaido University Hospital and 9 clinical staff members at Hokkaido University. Serum was collected from the recipients and controls after the third dose of the SARS-CoV-2 vaccine. The study protocol was approved by the Institutional Review Board of Hokkaido University Hospital (approval number: 022-0217). This study was conducted following the principles of the Declaration of Helsinki, 1996. Written informed consent was obtained from all the patients and healthy volunteers.

### Measurement of anti-SARS-CoV-2 spike protein IgG antibody

Serum samples were collected, and anti-SARS-CoV-2 spike protein IgG and anti-N IgG antibodies were measured using a fully automated chemiluminescent enzyme immunoassay (CLEIA). The CLEIA was performed using SARS-CoV-2 S-IgG (IB) reagents (Fujirebio, Tokyo, Japan) and a Lumipulse L2400 system (Fujirebio). Anti-SARS-CoV-2 spike protein IgG antibody titers were measured at SRL (Tokyo, Japan), with a cutoff value set at 1.0 AU/mL. We defined KTXRs whom IgG antibody titers were ≧1.0 AU/mL as responders, and IgG antibody titers were < 1.0 AU/mL as nonresponders.

### Cell culture

TMPRSS2-expressing Vero E6 (VeroE6/TMPRSS2) cells were obtained from the Japanese Collection of Research Bioresources Cell Bank (JCRB1819) and cultured in low-glucose Dulbecco’s modified Eagle’s medium (DMEM; Sigma-Aldrich, St. Louis, MO, USA) containing 10% fetal bovine serum (FBS) (Biowest, Bradenton, France) and G418 (Nacalai Tesque, Kyoto, Japan). VeroE6/TMPRSS2 cells were cultured at 37 °C under 5% CO_2_.

### SARS-CoV-2 reverse genetics

Recombinant SARS-CoV-2 was generated using a circular polymerase extension reaction (CPER) as previously described^58^. Briefly, nine DNA fragments encoding the partial genome of SARS-CoV-2 (strain 2019-nCoV/Japan/TY/WK-521/2020, GISAID ID: EPI_ISL_408667) were amplified with polymerase chain reaction (PCR) using PrimeSTAR GXL DNA polymerase (Takara Bio Inc., Shiga, Japan) and primers sets in the Table S1. A linker fragment encompassing the hepatitis delta virus ribozyme, bovine growth hormone poly A signal, and cytomegalovirus promoter was also prepared using PCR. Ten obtained DNA fragments were mixed and used for CPER^58^. To prepare GFP-expressing replication-competent recombinant SARS-CoV-2, we used fragment 9 (F9), where the sfGFP gene was inserted into the ORF7a frame instead of the authentic F9^58^. The rBA.1 S-GFP virus was provided by K. Sato at Tokyo University^59^ and the rBA.5 S-GFP virus was previously generated^11^. To generate rXBB.1.5-GFP, rEG.5.1-GFP, and rBA.2.86-GFP, nine DNA fragments encoding the partial genome of SARS-CoV-2 XBB.1.5 (strain TKYmbc30523/2022, GISAID ID: EPI_ISL_16697941), EG.5.1 (strain KU2023071028, GISAID ID: EPI_ISL_18072016), BA.2.86 (strain TKYnat15020, GISAID ID: EPI_ISL_18233521) were amplified with polymerase chain reaction (PCR) using PrimeSTAR GXL DNA polymerase (Takara Bio Inc., Shiga, Japan). sfGFP was inserted into the ORF7a frame of the corresponding each F9 fragment by inverse fusion PCR cloning. Then, these nine fragments and a linker fragment were mixed and used for CPER^58^. The CPER product was transfected into VeroE6/TMPRSS2 cells using TransIT-X2 Dynamic Delivery System (Takara) according to the manufacturer's protocol. At 5–8 days post-transfection, the culture medium was harvested and centrifuged, and the supernatants were collected as seed viruses. Nucleotide sequences were determined using a DNA sequencing service (Fasmac, Kanagawa, Japan), and sequence data were analyzed using ApE. We have confirmed that the recombinant SARS-CoV-2 can infect cells and the virus particles are released from the infected cells to the culture supernatant (Fig. S2).

### SARS-CoV-2 preparation and titration

The recombinant SARS-CoV-2 was amplified in VeroE6/TMPRSS2 cells, and the culture supernatants were harvested and stored at − 80℃ until use. Infectious titers in the culture supernatants were determined using 50% tissue culture infective doses (TCID50). The TCID50 was calculated using the Reed-Muench method. The culture supernatants of the cells were inoculated onto VeroE6/TMPRSS2 cells in 96-well plates after serial tenfold dilution with low-glucose DMEM containing 2% FBS and 1 mg/mL G418, and the infectious titers were determined 96 h post-infection. All experiments involving SARS-CoV-2 were performed in biosafety level-3 laboratories following standard biosafety protocols approved by Hokkaido University.

### Neutralizing antibody titer assay

In each well, 7.5 × 10^3^ VeroE6/TMPRSS2 cells were seeded in 96-well plates and maintained in DMEM (high glucose) containing 10% FBS and 1% PS. The cells were then incubated overnight. Each serum sample was serially diluted three-fold in the culture medium the following day, with a first dilution of 1:10 (final dilution range of 1:21,870). The diluted serum was incubated with 700 TCID50 of the chimeric recombinant SARS-CoV-2 at 37 ℃ in 5% CO_2_ for 1 h. Then, the mixture of chimeric recombinant SARS-CoV-2 and serum was added to VeroE6/TMPRSS2 cells in a 96-well plate. After 1 h, the cells were washed with DMEM (high glucose) containing 10% FBS and 1% PS.

In the case of using GFP fluorescence assay, after incubating the plates at 37 °C for 34–36 h (rWT S-GFP, rBA.1 S-GFP, rBA.5 S-GFP, rXBB.1.5-GFP, rEG.5.1-GFP) and 82–84 h (rBA.2.86-GFP), GFP fluorescence was detected using ECLIPSE Ts2 (Nikon, Tokyo, Japan). Subsequently, the luminance of GFP was calculated using Image J. A GFP signal with a luminance value > 150 in one field of view was considered positive. The neutralizing antibody titer was defined as the minimum serum dilution at which the GFP signal was positive. The neutralizing antibody titer of each serum sample was defined as the common logarithm (log10) of the average titer of the neutralizing antibody in triplicate assays. Data were plotted using GraphPad Prism 9 software (GraphPad Software, MA, USA).

### Analysis of SARS-CoV-2 spike-specific T cells

Heparin-treated whole blood was drawn into Gebrauchsanleitung Leucosep (Greiner Bio-One) and processed to isolate PBMCs according to the manufacturer’s instructions. Isolated PBMCs were cryopreserved in FBS containing 10% dimethyl sulfoxide (Sigma-Aldrich) and stored in − 196 °C liquid nitrogen until used in the assays. For analyzing SARS-CoV-2 spike-specific T cells, we performed surface and intracellular cytokine staining of CD4 and CD8 T cells as described previously^14,15^. Briefly, PBMCs were incubated in 1 mL RPMI 1640 medium containing 50 U/mL benzonase nuclease (Millipore, Darmstadt, Germany), 10% FBS, and PS for 1 h. Subsequently, the cells were incubated in 200 µL medium with or without peptides (17-mers overlapping by 10 residues) corresponding to the full-length SARS-CoV-2 spike (Wuhan-1, BA.1, or BA.5) at a final concentration of 2 µg/mL of each peptide, for 30 min. Spike peptide pools were prepared by mixing the individual peptides, which were synthesized by Eurofins, Japan. Each peptide pool contains a total of 181 peptides. Peptide pools corresponding to each variant were prepared individually. Thereafter, 0.2 µL BD GolgiPlug and 0.14 µL BD GolgiStop (both from BD Biosciences) were added and incubated for 5.5 h. The cells were subsequently stained using a LIVE/DEAD Fixable Blue Dead Cell Stain Kit (Thermo Fisher Scientific) and anti-CD3 (SP34-2), anti-CD8 (RPA-T8), anti-CD4 (L200), anti-CD45RO (UCHL1), and anti-CD27 (O323) antibodies. After fixation and permeabilization using the Cytofix/Cytoperm kit (BD Biosciences), the cells were stained with anti-CD154 (TRAP1) and anti-IFN-g (4S.B3), anti-TNF (MAb11), anti-IL-2 (MQ-17H12), anti-IL-4 (8D4-8), and anti-IL-13 (JES10-5A2). Analysis was conducted using a BD FACSymphony A5 flow cytometer (BD Biosciences). Data were interpreted using FlowJo v. 10.8.1. After gating live single T cells based on the forward scatter area and height (FSC-A and -H), side scatter area (SSC-A), live/dead cell exclusion, and CD3 staining, we separated live single T cells into CD4 + and CD8 + T cells. Subsequently, CD4 + and CD8 + T cells were further classified into non-naïve phenotypes based on their CD27 and CD45RO expression levels (Fig. S3). We defined CD154 + CD4 + T cells expressing IFN-g, TNF, or IL-2 as Th1 cells and those expressing IL4 or IL-13 as Th2 cells^12–14^. For spike-specific CD8 + T cells, non-naïve cells were gated based on IFN-g or TNF expression. The frequency of cytokine production was determined by background subtracting the DMSO-supplemented group as a control.

### Statistical analysis

The association between categorical variables was tested using the Chi-square test. Fisher’s exact test was used when appropriate. Statistical significance between the independent and dependent nonparametric data groups was evaluated using the Mann–Whitney *U* and Wilcoxon signed-rank test, respectively. Dunn’s multiple-comparison method was used to test for significant differences among the three groups. Pearson’s coefficient was used for the correlation analysis. Statistical significance was set at P < 0.05. GraphPad Prism 8.0.0 (GraphPad Software Inc., San Diego, CA, USA) was used for all analyses.

### Supplementary Information

Supplementary Figure S1.

Supplementary Figure S2.

Supplementary Figure S3.

Supplementary Legends.

Supplementary Table S1.

Supplementary Table S2.

Supplementary Table S3.

## Data Availability

All data generated or analyzed during this study are included within the manuscript or supplementary information. Further enquiries can be directed to the corresponding author.
