# Supplementary figures and images for: Neutralizing antibody responses and cellular responses against SARS-CoV-2 Omicron subvariants after mRNA SARS-CoV-2 vaccination in kidney transplant recipients
Source: Sci Rep. 2024 May 28;14:12176. doi: 10.1038/s41598-024-63147-z (PMC11133393; doi:10.1038/s41598-024-63147-z)

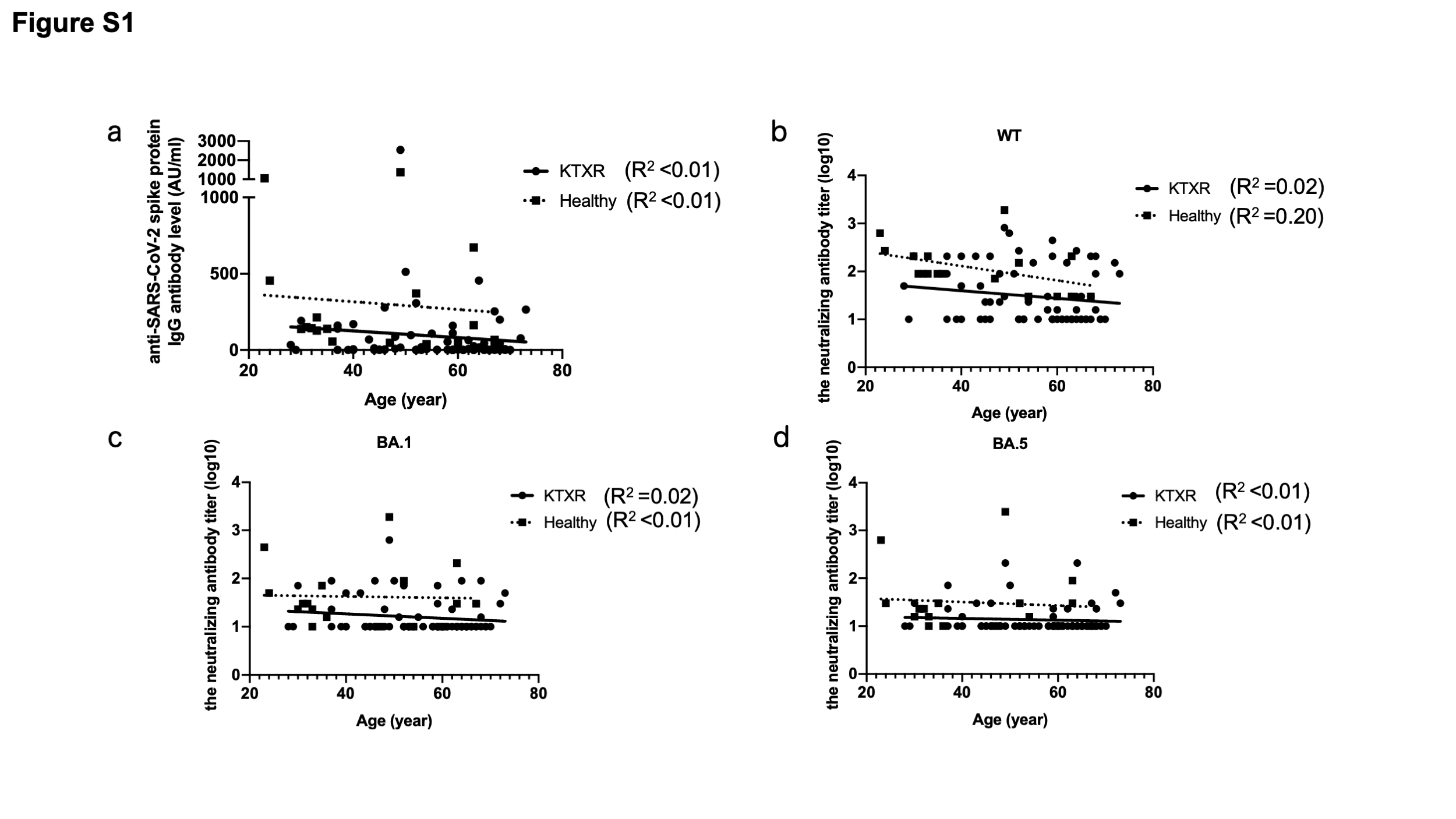

Supplement: Supplementary file 1 — Supplementary Figure S1. [file 41598_2024_63147_MOESM1_ESM.tiff]

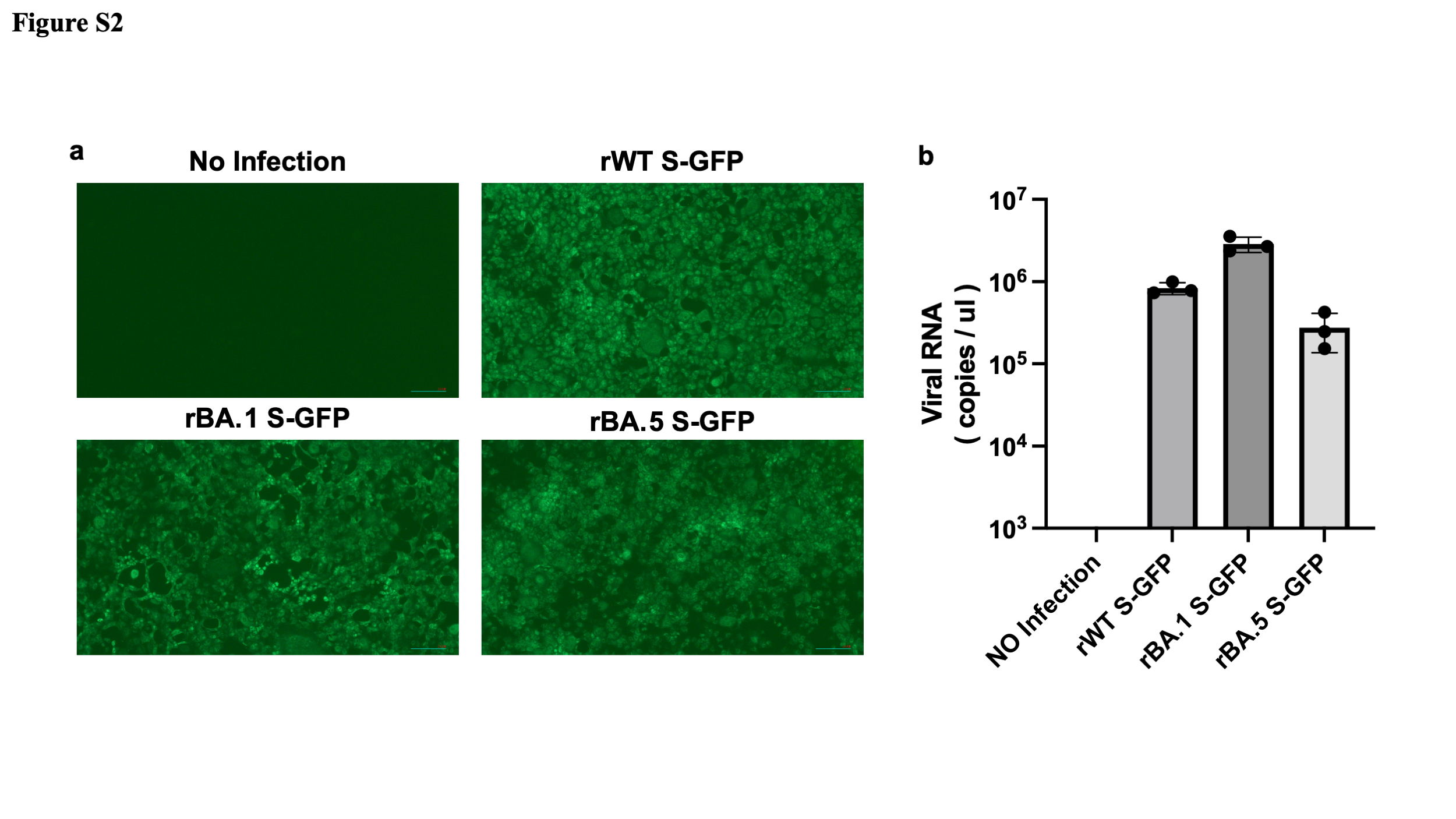

Supplement: Supplementary file 2 — Supplementary Figure S2. [file 41598_2024_63147_MOESM2_ESM.tiff]

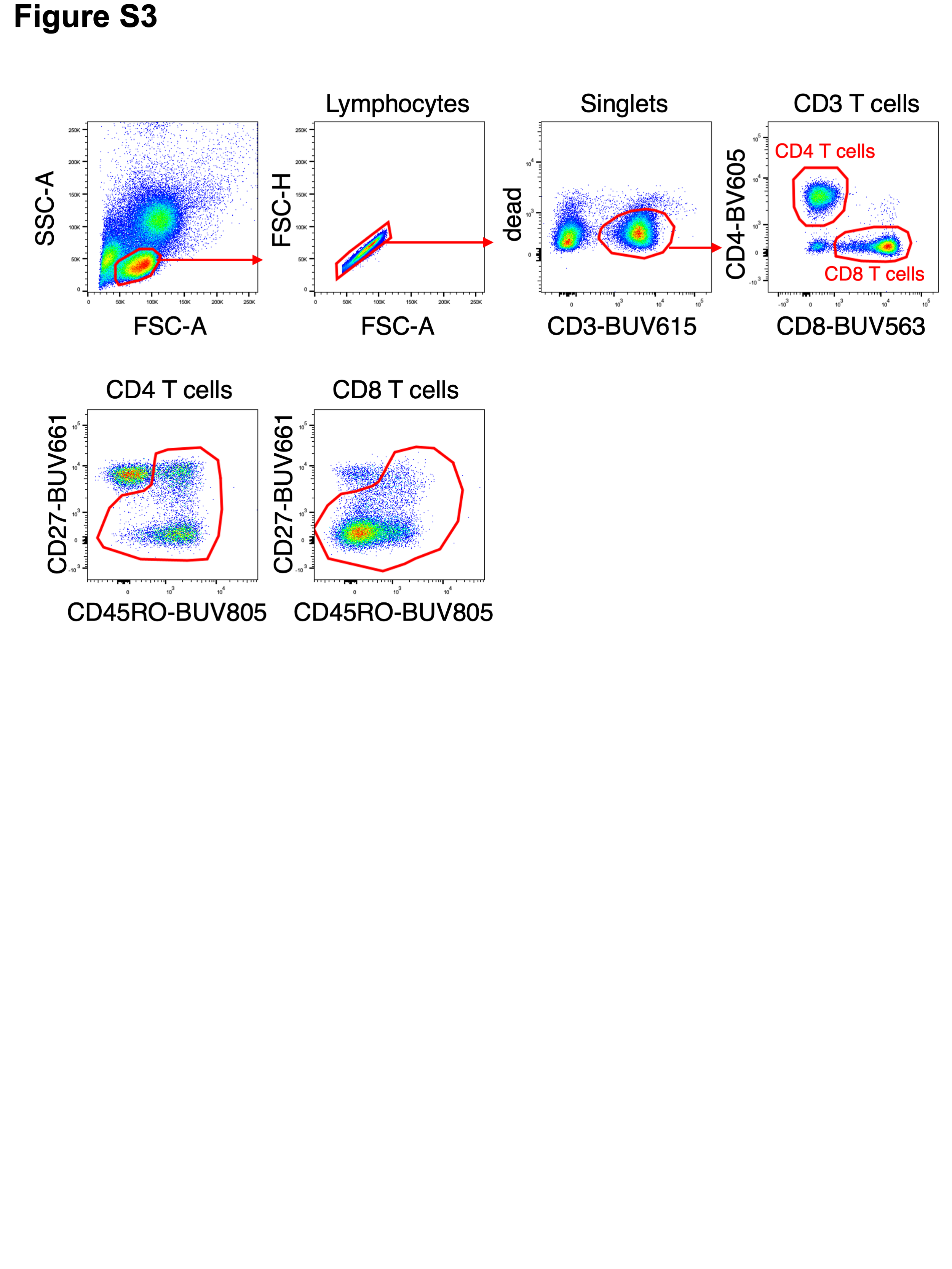

Supplement: Supplementary file 3 — Supplementary Figure S3. [file 41598_2024_63147_MOESM3_ESM.tiff]
