# Supplementary material for: Neutralizing antibody responses and cellular responses against SARS-CoV-2 Omicron subvariants after mRNA SARS-CoV-2 vaccination in kidney transplant recipients
Source: Sci Rep. 2024 May 28;14:12176. doi: 10.1038/s41598-024-63147-z (PMC11133393; doi:10.1038/s41598-024-63147-z)
Supplement: Supplementary file 4 — Supplementary Legends. [file 41598_2024_63147_MOESM4_ESM.docx]

Supplementary figure legends

Figure S1: Correlation between age and (a) anti-SARS-CoV-2 spike protein IgG antibody titers, (b, c, d) the neutralizing antibody titer against (b) WT, (c) BA.1 and (d) BA.5) .

Age is not correlated with anti-SARS-CoV-2 spike protein IgG antibody titers or neutralizing antibody titers.

Figure S2: The quality check of GFP-carrying recombinant SARS-CoV-2 with spike protein of WT, BA.1, or BA.5 generated by reverse genetics.

(a and b) VeroE6/TMPRSS2 cells were infected with these viruses (MOI=0.1). After 36 hours post-infection, the expression of GFP in VeroE6/TMPRSS2 cells was observed by florescent microscopy (a) and the copy number of the viral RNA in the cellular supernatants were determined by RT-qPCR (b).

Figure S3 Gating strategy for antigen-specific CD4^+^ and CD8^+^ T-cells.

After gating live single T-cells, based on forward scatter area and height (FSC-A and -H), side scatter area (SSC-A), live/dead cell exclusion, and CD3 staining, we separated the peripheral blood mononuclear cells (PBMCs) into CD4^+^ and CD8^+^ T-cells. Subsequently, CD4^+^ and CD8^+^ T-cells were further divided into non-naïve phenotypes based on the expression of CD27 and CD45RO.
